# Epistasis and genotype-by-environment interaction of grain protein content in durum wheat

**DOI:** 10.1590/S1415-47572010000100021

**Published:** 2010-03-01

**Authors:** Fethi Bnejdi, Mohamed El Gazzah

**Affiliations:** Laboratoire de Génétique et Biométrie, Département de Biologie, Faculté des Sciences de Tunis, Université Tunis El ManarTunisia

**Keywords:** epistasis, genotype x environment interaction, heritability, grain protein, wheat

## Abstract

Parental, F_1_ , F _2_ , BC _1_ and BC _2_ generations of four crosses involving four cultivars of durum wheat (*Triticum durum* Desf.) were evaluated at two sites in Tunisia. A three-parameter model was found inadequate for all cases except crosses Chili x Cocorit 71 at site Sidi Thabet and Inrat 69 x Karim at both sites. In most cases a digenic epistatic model was sufficient to explain variation in generation means. Dominance effects (h) and additive x additive epistasis (i) (when significant) were more important than additive (d) effects and other epistatic components. Considering the genotype-by-environment interaction, the non-interactive model (m, d, h, e) was found adequate. Additive variance was higher than environmental variance in three crosses at both sites. The estimated values of narrow-sense heritability were dependent upon the cross and the sites and were 0%-85%. The results indicate that appropriate choice of environment and selection in later generations would increase grain protein content in durum wheat.

## Introduction

Durum wheat (*Triticum durum* Desf.) is the most important cereal crop in Tunisia and North Africa, and is used primarily for couscous, macaroni and various types of bread (Troccoli *et al.*, 2000). In addition, wheat of high grain protein content usually commands a premium price. The grain protein content of durum wheat is an important trait for the nutritional value of grain and the technological properties of flour (Blanco *et al.*, 2006). The unpredictability of the Mediterranean climate causes fluctuations in wheat yield and quality, but offers the opportunity for obtaining high-quality durum wheat in terms of grain protein content (Rharrabti *et al.*, 2001). The higher-yielding cultivars of Tunisia tend to have low grain protein, whereas the lower-yielding cultivars tend to have high grain protein. This inverse relationship between wheat yields and grain protein content is well known (Terman *et al.*, 1969; Entz and Fowler, 1989; Pleijel *et al.*, 1999). Genetic differences and environmental effects on grain protein content have been reported previously (Kramer, 1979; Baenziger *et al.*, 1985). A range of heritabilities for grain protein content have been found in bread wheat: 47%-83% (Ekiz *et al.*, 1998); 39%-61% (Guthrie *et al.*, 1984); 30%-76% with a mean of 44% (Duffield *et al.*, 1972). Many data indicate that in wheat the grain protein content is heritable and determined either by several genes (Johnson *et al.*, 1968) or by one or two genes (Haunold *et al.*, 1962; Cowley and Wells, 1980). Millet *et al.* (1992) concluded that grain protein content was largely determined by the maternal parent. The estimation of epistatic components of genotypic variance and genotype x environment interaction is unusual in genetic studies, as epistasis was considered to make only a small contribution to quantitative variation (Crow, 1987). However, recent studies indicate the contribution of epistatic effects and genotype x environment interaction to grain protein content in barley (Kaczmarek *et al.*, 2002). In this study, a generation mean analysis methodology (Mather and Jinks, 1982) was used to estimate the inheritance of protein content in durum wheat*.* This method allows determining whether the protein content traits fit an additive-dominance model and estimating the additive, dominance, and epistatic gene effects, as well as the environmental effects and the genotype x environment interaction (Mather and Jinks, 1982).

## Materials and Methods

The study was carried out under rain-fed conditions at two locations in Tunisia (Mogran and Sidi Thabet), during the years 2005-2006. Sowing was done at the beginning of December. The Mogran area is characterized by loam soil and a sub-humid climate with an annual rainfall of about 700 mm. The Sidi Tahbet area is also characterized by loam soil and a humid climate with an annual rainfall of about 400 mm. Parental cultivars were selected for their differences in grain protein content. Plants were grown in a randomised complete block design with two replications, with a between-row spacing of 20 cm and a within-row spacing of 10 cm. Harvest was done per plant, and the numbers of plants evaluated varied depending on the generation: in generations with greater segregation, such as F_2_, BC_1_ and BC_2_, a greater number of plants were evaluated. The grain protein content was assessed by Near-Infrared Reflectance Spectroscopy of grain flour of each individual plant, using an Inframatic 8600 flour analyser. Transforming the data by log, square root, arc-sine, and arc-sine of square root had no effect on data distribution or in removing epistatic effects. Analyses of variance by population and location using SAS software version 6 (SAS Institute, 1990) indicated that the replication and generation x replication effects were not significant. Therefore, the generation mean analyses were made without adjusting the data for replication.

Calculated means and variances were used to estimate the mid-parent (m), additive (d), and dominance (h) gene effects, as described by Rowe and Alexander (1980) following the method of Mather and Jinks (1982) for a three-parameter model. Adequacy of the additive-dominance model was determined by the chi-square (*X*^*2*^) test with three degrees of freedom and was accepted if p > 0.05 (non-significant *X*^*2*^ value). When the three-parameter model was inadequate (significant *X*^*2*^ value), the interaction terms [additive x additive (i), additive x dominance (j), and dominance x dominance (l)] were computed (Mather and Jinks, 1982). The genetic parameters [m, (d), (h), (i), (j), and (l)] were tested for significance using an unpaired *t*-test. Adequacy of the best fit model was determined by the *X*^*2*^ test with three degrees of freedom and was accepted if p > 0.05 (non-significant *X*^*2*^ value). The weighted least squares method was also used to estimate environmental and genotype x environment interactions. This method was applied to parents and F_1_ only (Mather and Jinks, 1982).

## Heritability

The homogeneity of variances of non-segregating generations was tested using Bartlett's test (Bartlett, 1937). When the variances were heterogeneous, the environmental variance (V_E_) was replaced by an adequate number of separate parameters and pooled to produce a single environmental variance. Additive, dominance, additive x dominance and environmental variance components were estimated using the maximum likelihood method with the observed variance of the six basic generations used as the initial weights (df /2*S^2^) until the *X*^*2*^ test value reached a minimum (Lynch and Walsh, 1998).

Narrow-sense heritability (h^2^_n_) was calculated as follows: h^2^_n_ = V*_A_/V*_A_ + V*_D_ + V_E_, where V*_A_ is the additive genetic component of variance, V*_D_ the dominance genetic component of variance, and V_E_ the environmental variance (Kearsey and Pooni, 1996).

## Results

The mean values and variances for the analysed traits of the four crosses at the two sites are presented in Table 1. In all cases, depending on the site, the means of the parents in each cross showed a tendency to be more extreme. The backcrosses BC_1_P_1_ and BC_1_P_2_ had means that tended to be close to those of their respective recurrent parents. These results confirmed the choice of parents for the present study. The F_1_ means exceeded the superior parents for crosses Inrat 69 x Cocorit 71 and Chili x Karim at the Sidi Thabet site and for Inrat 69 x Cocorit 71 at Mogran. In the majority of cases, the F_2_ mean was higher than the P_2_ mean and lower than the F_1_ mean.

The results of the three-parameter model and the best-fit model are listed in Table 2. The joint scaling test revealed that the additive-dominance model was adequate in three cases; in other cases it was inadequate (p < 0.001). The failure of the model may be due to the influence of interaction or linkage among genes governing the inheritance of this trait. Therefore, the digenic epistatic model was applied and was found adequate; this adequateness ranged from 2 to 98%. Additive (d) and dominance (h) effects were significant in the majority of crosses. For the crosses Chili x Cocorit 71 at Mogran, and Inrat 69 x Karim at both Mogran and Sidi Thabet, protein content was adequately explained by an additive-dominance model, with the additive effect being more important than the dominance effect. For all other cases a digenic epistatic model was adequate. The additive effect was significant and positive in all crosses at the two sites. The dominance effect was not significant only for crosses Inrat 69 x Karim at Mogran and Chili x Karim at Sidi Thabet. For the digenic epistatic effect, the additive x additive (i) effect was significant in the majority of cases; the dominance x dominance (l) effect was significant in one case, and the additive x dominance (j) effect was significant in two cases. Generation mean analysis of the non-segregating generations in the present study showed that the estimates of environment x dominance and of environment x additive effects were not significantly different from zero in all crosses, and the four-parameter models were fitted (Table 3).

The estimates of the components additive variance, dominance variance, environmental variance and h^2^_n_ are presented in Table 4. For the cross Chili x Cocorit 71, environmental variance was higher at Mogran, additive variances were more pronounced than all other components, and their values were 8.84-9.28. Dominance variances were negative and not significant. The values of h^2^_n_ varied, depending on the testing site, ranging from 64%-85%. For the Inrat 69 x Karim cross, the environmental variances were similar at both sites. Additive variances were more pronounced at Mogran, with a range of 5.86-6.43. At both sites, h^2^_n_ was similar, with a range of 50%-53%. For Inrat 69 x Cocorit 71, the environmental variance range was 7.07-8.3. Additive variances were negative. Therefore, h^2^_n_ was not estimated. For the cross Chili x Karim, the environmental variance was 2.00-3.83. Additive variances were more pronounced at Mogran, with a range of 2.6-4.45. Heritability was similar between the sites, ranging from 48%-53%.

## Discussion

In all four populations, the means of the parents (P_1_ and P_2_) had a tendency to be more extreme and contrasting than the means of the F_1_ and F_2_ generations. The backcrosses BC_1_P_1_ and BC_1_P_2_ had means that tended to be similar to those of their respective recurrent parents. These results confirmed that the choices of parents for the present study were contrasting, which is a prerequisite for generation means analysis, as proposed by Mather and Jinks (1982). Generation means analysis has been used to study the inheritance of other complex traits in wheat, such as resistance to yellowberry (Bnejdi and El Gazzah, 2008), carbon isotope discrimination (Rebetzke *et al.*, 2006), spike length (Sharma *et al.*, 2003), plant height, number of heads per plant, number of grains per spike and grain yield per plant (Novoselovic *et al.*, 2004).

Depending on the cross and experimental site, in most cases the variation in the generation means fitted a digenic epistatic model. This finding indicated that improvement of grain protein content would be moderately difficult compared to a situation in which an additive-dominance model is a better fit, and more favourable than for a tri-genic epistatic effect. The results agree with those of Kraljevic-Balalic *et al.* (1982), who found that, for grain protein content in bread wheat, most of the variation was due to additive and non-additive genetic variation. Similar results were reported by Ketata *et al.* (1976) and Joshi *et al.* (2004) in *Triticum aestivum*. An additive effect only was reported by Chapman and McNeal (1970) and Zahid *et al.* (2007).

The results of the present study revealed the limitation of most quantitative genetic studies based on the assumption of negligible epistasis. Many cases of significant epistasis have been reported for this trait in barley (Kaczmarek *et al.*, 2002) and bread wheat (Kraljevic-Balalic *et al.*, 1982).

For the crosses Chili x Cocorit 71 at Sidi Thabet and Inrat 69 x Karim at Mogran and Sidi Thabet, the additive-dominance models were adequate. This indicated that the mode of gene action was site-dependent. The presence or absence of epistasis may depend upon the environment in which the plant material was evaluated and thus may not always be related to the inherent capacity of a genotype (Sunil and Singh, 2003). Kumar *et al.* (2003) reported that the genetic system governing grain protein content was highly additive. When an additive-dominance model was adequate, the magnitude of the additive (d) gene effect was greater than those of dominant (h) gene effects, indicating the major role of additive gene effects compared to dominance effects in controlling variation in grain protein content. With respect to epistatic effects, additive x additive effects were significant in all cases when a di-genic model was applied. Dominant x dominant effects (l) were significant only for one case, and dominant x additive (j) effects only for two cases. This situation is more favourable than the presence of dominant x dominant and dominant x additive effects in all cases. Epistasis of the additive x additive (i) type as observed in this experiment could be exploited in a breeding programme with the additive component, since it can be fixed by selection. Nevertheless, the additive x dominance (j) and dominance x dominance (l) types of interactions may not be an advantage in developing inbred varieties, as these are not fixable by selection.

Generation means analysis of genotype and genotype x environment interaction indicated that the non-interactive model was fitted. This situation is more favourable than the presence of environment x dominance and environment x additive effects.

Maximum likelihood estimates of environmental variance were higher at Mogran than at Sidi Thabet. The additive variance component was not consistent between crosses and sites and was higher for the cross Chili x Cocorit 71. The dominance variance component varied between crosses and sites.

Our results showed h^2^_n_ values which were moderate to high, suggesting a large participation of the genetic effects on phenotypic expression of grain protein content, and also that selection for the trait should be highly efficient. These results are similar to those reported by Ekiz *et al.* (1998) and Sharma and Sharma (2007). From a breeder's point of view, the h^2^_n_ estimates from the two sites show that the Chili x Cocorit 71 cross has the greatest chance of genetic improvement. Selections in later generations with increased homozygosity, where additive and additive x additive variances are established, are recommended. For further increase in the grain protein content of durum wheat, it is suggested that an appropriate environment should be chosen, so that the character will show relatively simple inheritance.

## Figures and Tables

**Table 1:** Plant means ± SE for grain protein (%) in parental and offspring populations from four crosses at two sites (Mogran and Sidi Thabet), with two replications.

Population	Chili x Cocorit 71	Inrat 69 x Karim	Inrat 69 x Cocorit 71	Chili x Karim
	Sidi Thabet			
P_1_	16.60 ± 1.24a (**20**)^y^	15.04 ± 2.78a (**20**)	15.04 ± 2.78ab (**20**)	16.60 ± 1.24a (**20**)
BC_1_P_1_	15.33 ± 2.74a (**50**)	14.30 ± 2.73a (**50**)	14.76 ± 3.54b (**48**)	15.92 ± 1.89a (**47**)
F_1_	16.58 ± 1.13a (**19**)	14.57 ± 2.26a (**26**)	16.50 ± 2.48a (**22**)	17.00 ± 1.53a (**19**)
F_2_	11.22 ± 2.81b (**96**)	13.66 ± 3.23ab (**98**)	11.31 ± 3.02c (**94**)	13.73 ± 2.31b (**97**)
BC_1_P_2_	11.07 ± 1.68b (**53**)	12.32 ± 2.76bc (**50**)	11.05 ± 2.88c (**48**)	13.43 ± 2.52b (**46**)
P_2_	11.40 ± 1.98b (**20**)	11.45 ± 2.39c (**20**)	11.40 ± 1.98c (**20**)	11.45 ± 2.39c (**20**)
	Mogran			
P_1_	12.60 ± 1.99a (**20**)	11.80 ± 2.15a (**20**)	11.80 ± 2.15a (**20**)	12.60 ± 1.99a (**20**)
BC_1_P_1_	12.13 ± 3.57ab (**52**)	11.26 ± 3.21a (**50**)	11.31 ± 3.30ab (**48**)	12.22 ± 2.37a (**37**)
F_1_	12.47 ± 3.28a (**19**)	10.46 ± 3.02ab (**26**)	12.72 ± 2.18a (**22**)	12.40 ± 1.70a (**23**)
F_2_	10.86 ± 3.72ab (**88**)	10.19 ± 3.57ab (**78**)	9.55 ± 3.16c (**84**)	10.44 ± 2.47ab (**98**)
BC_1_P_2_	10.46 ± 2.77b (**43**)	10.52 ± 2.99ab (**50**)	9.92 ± 3.32bc (**48**)	11.62 ± 1.74c (**46**)
P_2_	8.25 ± 2.29c (**20**)	9.10 ± 2.26b (**20**)	8.25 ± 2.29c (**20**)	9.10 ± 2.26b (**20**)

Means followed by the same letter within each column for each site are not significantly different, based on Duncan's test (p < 0.05). y = numbers in parentheses represent the plants evaluated in each generation.

**Table 2:** Estimates of gene effects ± SE for grain protein content in four crosses (Chili x Cocorit 71, Inrat 69 x Karim, Inrat 69 x Cocorit 71, and Chili x Karim) at two sites (Mogran and Sidi Thabet), with two replications.

	Sidi Thabet	Mogran		Sidi Thabet	Mogran
	Chili x Cocorit 71			Inrat 69 x Karim	
Model	Three-parameter model				
m	12.38 ± 0.23**	10.35 ± 0.34**		12.97 ± 0.34**	10.53 ± 0.32**
d	3.54 ± 0.23**	2.04 ± 0.32**		1.83 ± 0.33**	1.19 ± 0.30**
h	2.86 ± 0.38**	1.80 ± 0.71*		1.26 ± 0.61*	0.12 ± 0.63
^(A)^P	< 0.001	0.69		0.50	0.42

	Best-fit model				
m	5.79 ± 0.65**				
d	2.60 ± 0.27**				
h	10.76 ± 0.82**				
i	8.18 ± 0.73**				
l					
j	3.35 ± 1.04**				
^(A)^P	0.85				

	Inrat 69 x Cocorit 71			Chili x Karim	
Model	Three-parameter model				
m	12.27 ± 0.34**	9.58 ± 0.32**		13.62 ± 0.29**	10.74 ± 0.33**
d	1.94 ± 0.34**	1.74 ± 0.32**		2.7 ± 0.28**	1.45 ± 0.32**
h	2.07 ± 0.63**	2.33 ± 0.57**		2.31 ± 0.54**	1.43 ± 0.62**
^(A)^P	< 0.001	< 0.05		< 0.05	< 0.05

	Best-fit model				
m	6.04 ± 0.95**	6.46 ± 0.94**		10.49 ± 0.87**	8.96 ± 0.94**
d	1.81 ± 0.38**	1.71 ± 0.32**		2.56 ± 0.29**	1.75 ± 0.36**
h	10.39 ± 1.34**	6.29 ± 1.25**		6.52 ± 1.23	3.81 ± 1.34*
i	7.14 ± 1.04**	3.58 ± 1.02**		3.54 ± 0.93**	2.05 ± 1.02*
l					-2.40 ± 1.46*
j	3.85 ± 1.70*				
^(A)^P	0.69	0.87		0.98	0.02

Mean (m), additive (d), dominance (h), additive x additive (i), dominance x dominance (l), additive x dominance (j) genetic effects. *, **, indicate that means and gene effects are statistically different from zero at p < 0.05, 0.01, respectively. (A) = Probability of adequateness of model.

**Table 3:** Estimates of the genetic, environmental and genotype x environment interaction components (± SE) of generation means.

	Chili x Cocorit 71	Inrat 69 x Karim	Inrat 69 x Cocorit 71	Chili x Karim
m	12.22 ± 0.22**	11.86 ± 0.26**	11.61 ± 0.23**	12.44 ± 0.27**
d	2.44 ± 0.22**	1.56 ± 0.26**	1.81 ± 0.23**	2.13 ± 0.27**
h	2.45 ± 0.34**	0.78 ± 0.45	2.98 ± 0.36**	2.31 ± 0.45**
e	1.87 ± 0.20**	1.62 ± 0.22**	1.72 ± 0.18**	1.82 ± 0.22**
^(A)^P	0.56	0.24	0.72	0.08

Mean (m), additive (d), dominance (h) genetic effects; (e) environmental effect. *, **, indicate that means and gene effects are statistically different from zero at p < 0.05, 0.01, respectively. (A) = Probability of adequateness of model.

**Table 4:** Estimates of variance components ± SE and narrow-sense heritability (h^2^_n_) in four crosses at two sites (Mogran and Sidi Thabet), with two replications.

		V_E_	V_A_	V_D_	*X*^2^ (3df)	h^2^_n_
Chili x Cocorit 71						
	Sidi Thabet	1.55 ± 0.30**	8.84 ± 2.91**	-2.46 ± 1.73	s	0.85
	Mogran	5.08 ± 1.04**	9.28 ± 5.50*	-0.48 ± 3.79	ns	0.64

Inrat 69 x Karim						
	Sidi Thabet	5.79 ± 1.01**	5.86 ± 4.19	-1.16 ± 2.97	ns	0.50
	Mogran	5.52 ± 0.96**	6.43 ± 5.13	0.79 ± 3.60	ns	0.53

Inrat 69 x Cocorit 71						
	Sidi Thabet	7.07 ± 0.91**	-3.56 ± 4.88	4.02 ± 3.89	ns	0A
	Mogran	8.3 ± 0.11**	-3.86 ± 5.35	-48.35 ± 4.26	ns	0A

Chili x Karim						
	Sidi Thabet	2.00 ± 0.42**	2.6 ± 3.02	0.74 ± 2.18	ns	0.48
	Mogran	3.83 ± 0.81**	4.45 ± 3.28	-2.15 ± 2.34	ns	0.53

df = degrees of freedom, calculated as the number of generations minus the number of estimated variance parameters. ns = non-significant, s = significant. *, **, indicate that variance components are statistically different from zero at p < 0.05, 0.01, respectively. A = values assumed to be zero, due to negative estimates.
